# The LMO2 -25 Region Harbours GATA2-Dependent Myeloid Enhancer and RUNX-Dependent T-Lymphoid Repressor Activity

**DOI:** 10.1371/journal.pone.0131577

**Published:** 2015-07-10

**Authors:** Nicolas Bonadies, Berthold Göttgens, Fernando J. Calero-Nieto

**Affiliations:** Department of Haematology, Wellcome Trust and MRC Cambridge Stem Cell Institute, Cambridge Institute for Medical Research, Cambridge University, Cambridge, United Kingdom; Georg Speyer Haus, GERMANY

## Abstract

Lim domain only 2 (LMO2) is a transcriptional co-factor required for angiogenesis and the specification of haematopoietic cells during development. *LMO2* is widely expressed within haematopoiesis with the exception of T-cells. Failure to downregulate *LMO2* during T-cell maturation leads to leukaemia, thus underlining the critical nature of context-dependent regulation of *LMO2* expression. We previously identified a distal regulatory element of *LMO2* (element -25) that cooperates with the proximal promoter in directing haematopoietic expression. Here we dissected the functional activity of element -25 and showed it to consist of two modules that conferred independent and cell-type specific activities: a 3’ myeloid enhancer and a 5’ T-cell repressor. The myeloid enhancer was bound by GATA2 in progenitors and its activity depended on a highly conserved GATA motif, whereas the T-cell repressor moiety of element -25 was bound by the Core Binding Factor in T-cells and its repressive activity depended on a highly conserved RUNT motif. Since the myeloid enhancer and nearby downstream region is recurrently involved in oncogenic translocations, our data suggest that the -25 enhancer region provides an open chromatin environment prone to translocations, which in turn cause aberrant *LMO2*expression in T-cells due to the removal of the adjacent T-cell repressor.

## Introduction

Lim domain only 2 (LMO2) is a zinc-finger transcriptional co-factor required for angiogenesis and emergence of haematopoietic stem cells during ontogenesis [1–4]. LMO2 forms a complex with the LIM domain-binding protein 1 (LDB1) and the DNA-binding E-box and GATA transcription factors. This complex has been shown to be critical for the specification of haematopoietic stem cells and erythroid lineage [5–8]. *LMO2* was originally identified through its involvement in recurrent chromosomal translocations [9, 10]. *LMO2* is a major oncogene and its ectopic expression leads to T-cell lymphoproliferative disease and T-cell acute lymphoblastic leukaemia (T-ALL) [11–13]. Recently, gene-expression profiling studies revealed high *LMO2* expression in different subtypes of B-cell lymphoproliferative disorders or acute myeloid leukaemia, thus, suggesting a broader oncogenic effect in different haematopoietic lineages caused by failure of *LMO2* down-regulation [14–22]. Juxtaposition of TCR enhancers is thought to be the main driving mechanism for ectopic *LMO2* expression [23, 24]. However, this notion has recently been challenged by a detailed break point analysis in TCRdelta-LMO2 rearranged T-ALL patients [25]. Therefore, investigation of context-dependent regulation of important developmental genes such as *LMO2* remains instrumental for understanding oncogenic deregulation in leukaemogenesis.

The *LMO2* gene is localised on the short arm of chromosome 11 within band 13 (11p13) and its expression is tightly regulated in the haematopoietic system. *LMO2* expression is directed by a proximal and a distal promoter element that generate transcripts with distinct 5’ untranslated regions but an identical coding region derived from exons 3–6 [26]. Additionally, our group recently reported an intermediate promoter element (mdp) that mediates *LMO2* expression in a subset of T-acute lymphoblastic leukaemia patients [27]. We previously showed that the proximal promoter element of *LMO2* confers endothelial-specific activity [28], although additional distal regulatory elements are required for a comprehensive and context-dependent regulation of *Lmo2*-expression in haematopoiesis [29]. We identified a distal regulatory element located 25kb upstream of the proximal promoter (element -25) that cooperates with the proximal promoter in specifying cell-context dependent haematopoietic expression to foetal liver in transient transgenic mouse assays [29]. This element is highly enriched for acetylation of lysine 9 of histone H3 (H3K9ac), a histone mark associated with accessible regions of chromatin, in the haematopoietic progenitor cell line HPC7 and the myeloid progenitor cell line 416B [29]. Interestingly, medium to low levels of H3K9ac enrichment were found in endothelial (MS1), erythroid (MEL) and T- (BW5147) cell lines as well as whole adult murine thymus, suggesting dynamic occupancy of element -25 during differentiation. Moreover, when enhancer activity was tested in conjunction with the proximal promoter element in stable transfections, element -25 was the strongest from an array of 14 elements in the myeloid progenitor 416B cell line [29].

In the current study, we have dissected in detail the regulatory activity of element -25. We demonstrate that element -25 retains dual and cell-type specific activity, acting as a myeloid enhancer and a T-cell repressor. We defined a myeloid enhancer module in the 3’ region of element -25 that is dependent on a highly conserved GATA binding site and a T-cell repressor module localised in the 5’ region, which activity depends on a highly conserved RUNT binding site.

## Materials and Methods

### Cell lines and cell culture

The murine multipotent myeloid progenitor cell line 416B [30], erythroleukaemia cell line F4N [31], endothelial cell line MS1 [32] as well as the human T-cell lines Jurkat (DSMZ, ACC282) and Molt4 (DSMZ, ACC362) were cultured as previously described [27–29].

### Sequence analysis

Homologous genomic *LMO2* sequences of element -25 derived from human, mouse, cow, dog and cat were downloaded from *Ensembl*, aligned using *multi-Lagan* [33] and displayed using *Genedoc* [34]. Candidate transcription factor binding sites were identified using *Transcription Factor Binding Sites Search (TFBSsearch)* [35] and the *Transcription Element Search Software* (*TESS*: www.cbil.upenn.edu/tess *)* programs [36].

### Reporter constructs

The *LMO2 luciferase* reporter constructs were amplified from human genome using primers listed in S1 Table, cloned into pGL2-luciferase vectors (Promega Corporation, Madison, WI) and confirmed by sequencing. Deletion constructs were produced by restriction enzyme digestion and re-ligation. Mutation constructs were generated with QuickChange XL Site-Directed Mutagenesis Kit (Agilent, Santa Clara, CA) using primers listed in S2 Table. All constructs were confirmed by sequencing.

### Stable transfection experiments

**Fig 1:**
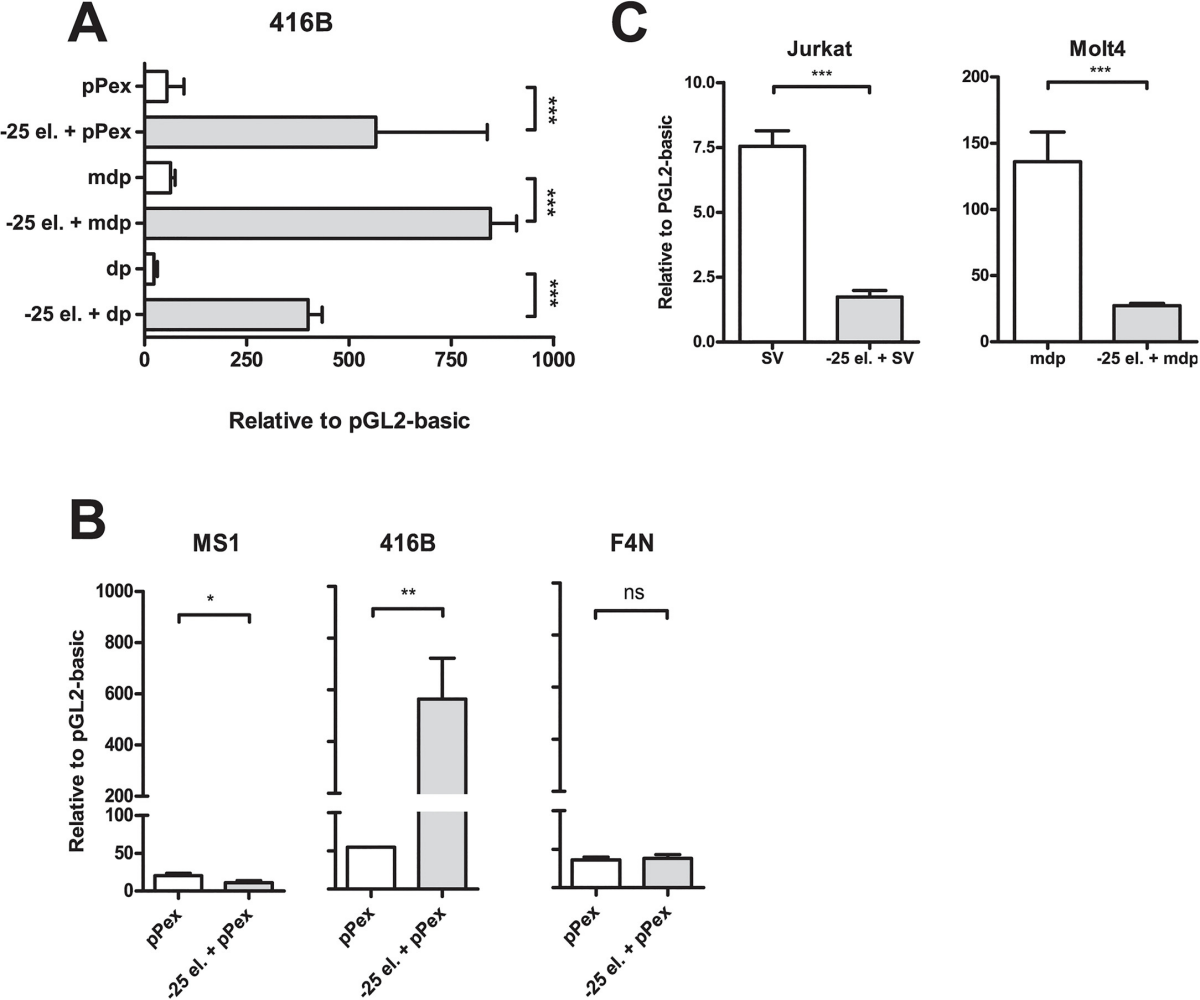
Cell-type specific activity of *LMO2* element -25. A) Promoter-independent enhancer activity of element -25 in multipotent myeloid progenitors 416B. Luciferase activity of *LMO2* proximal (pPex), intermediate (md) or distal (dp) promoter elements in the presence and absence of element -25 (-25 el.) was measured in 416B cells. B) Cell-type specific activity of -25kb DRE. Luciferase experiments were performed in endothelial MS1 and erythroid F4N cells using *LMO2* proximal (pPex) promoter element in the presence and absence of element -25 (-25 el.). For comparison purposes, 416B data from panel A is also shown. C) T-cell repressor activity of element -25. Luciferase experiments were performed in *LMO2* expressing Molt4 and *LMO2* non-expressing Jurkat cells, using, respectively, *LMO2* intermediate (md) or minimal SV promoter elements in the presence and absence of element -25 (-25 el.). In all cases, mean and standard error of the means (SEM) for at least two independent stable transfections (each one performed at least in triplicate) are shown. Values are expressed relative to empty vector, pGL2 basic. *P < 0.05; **P < 0.01; ***P < 0.001; ns, not significant.

**Fig 2:**
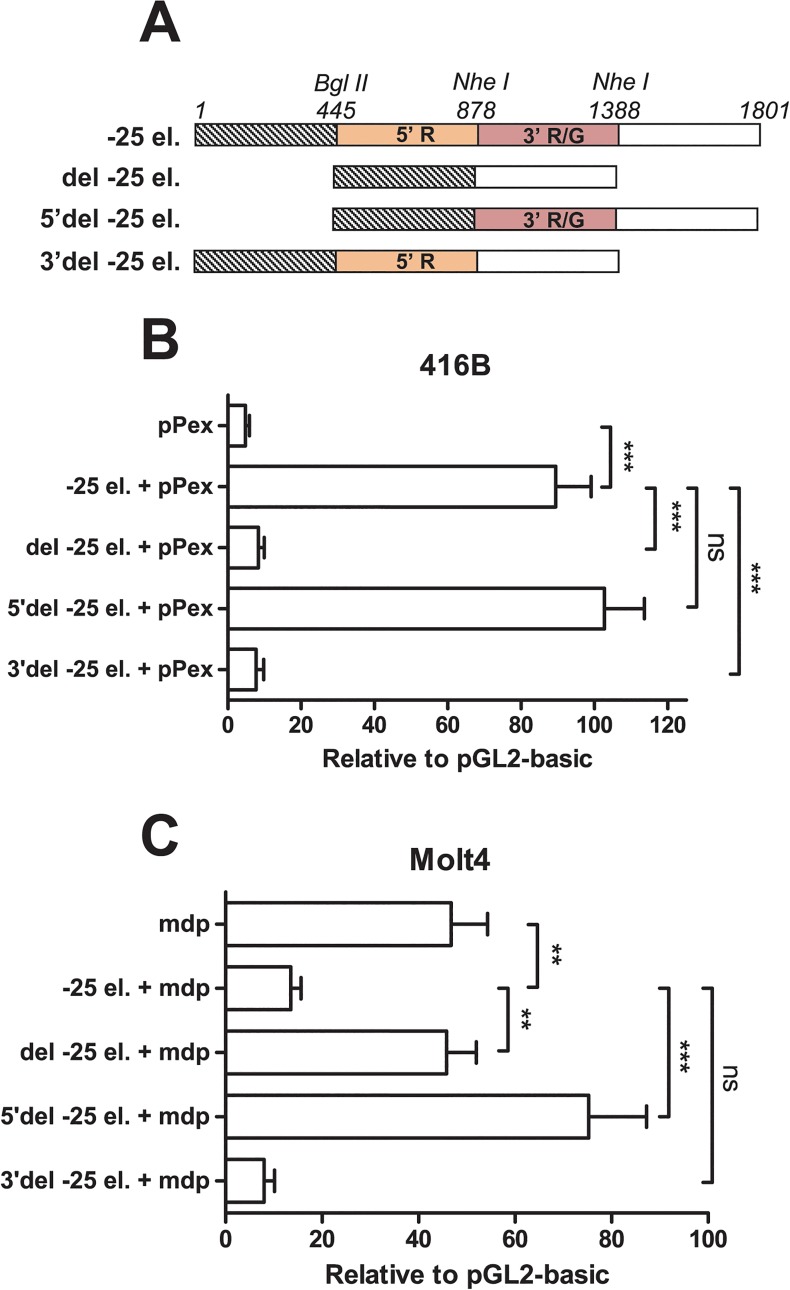
Dual myeloid enhancer and T-cell repressor activity of element -25. A) Diagram of element -25 and deletion constructs. 5’ T-cell repressor and 3’ myeloid enhancer modules of element -25 (-25 el.) are coloured in orange and red, respectively. The presence of the conserved 5’ RUNT (5’ R), 3’ RUNT and GATA (3’ R/G) motifs is indicated. Three deletion constructs (del -25 el., 5’del -25 el. and 3’del -25 el.) were produced by restriction enzyme digestion using *BglII* (position 445) and *NheI* (positions 878 and 1388). B, C) Independent myeloid enhancer and T-cell repressor activities of 2 modules within element -25. Deletion constructs were stably transfected together with either *LMO2* proximal (pPex) or intermediate (mdp) promoter elements in myeloid progenitors 416B (B) and T-cells Molt4 (C), respectively. In all cases, mean and standard error of the means (SEM) for at least two independent stable transfections (each one performed at least in triplicate) are shown. Values are expressed relative to empty vector, pGL2 basic. **P < 0.01; ***P < 0.001; ns, not significant.

**Fig 3:**
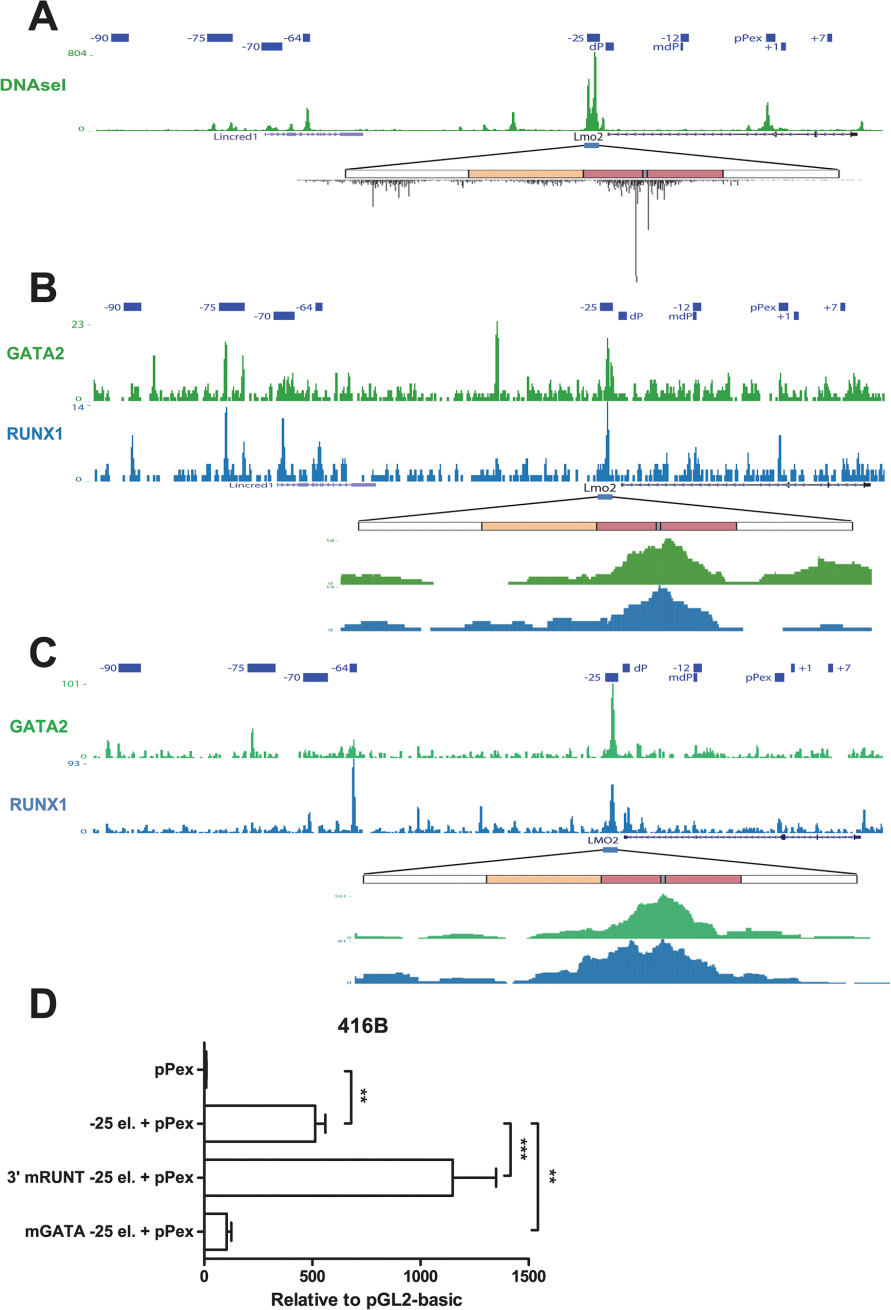
The 3’ myeloid enhancer activity of element -25 is dependent on GATA binding. A) DNaseI-Seq profile of *Lmo2* locus region in mouse myeloid progenitors 416B cells. Elements previously described to be involved in the regulation of *Lmo2* expression are indicated. The strongest DHS in the *Lmo2* locus co-localises with element -25. A magnification of the region corresponding to element -25 is shown. Diagram of element -25 as in Fig 2A. DNaseI profile shows a strong protection in the region corresponding to conserved GATA motif (location shown in grey). B, C) ChIP-Seq profile of RUNX1 and GATA2 in *LMO2* locus region in murine haematopoietic progenitors HPC7 (B) and human CD34+ (C) cells. Elements previously described to be involved in the regulation of *LMO2* expression are indicated. Strong binding for GATA2 and RUNX1 can be detected at element -25. A magnification of the region corresponding to element -25 is shown. Diagram of element -25 as previously. Binding of RUNX1 and GATA2 specifically takes place at the 3‘ myeloid enhancer module of element -25. D) Myeloid enhancer activity depends on a conserved GATA motif. Conserved RUNT and GATA motifs present in the myeloid enhancer module of element -25 were mutated (3’mRUNT -25 el. and mGATA -25 el., respectively). Mutated constructs were stably transfected together with *LMO2* proximal promoter element (pPex) in myeloid progenitor 416B cells and luciferase activity was measured. Mean and standard error of the means (SEM) for at least two independent stable transfections (each one performed at least in triplicate) are shown. Values are expressed relative to empty vector, pGL2 basic. **P < 0.01; ***P < 0.001.

**Fig 4:**
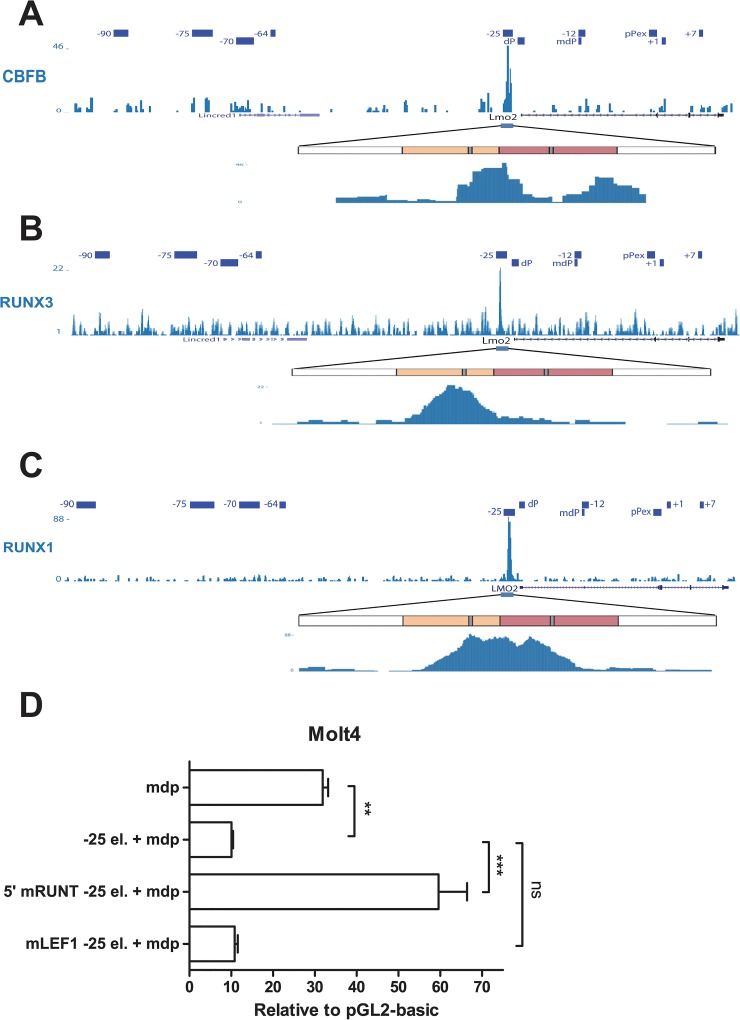
The 5’ T-cell repressor activity of element -25 is dependent on Runx binding. A, B, C) ChIP-Seq profile of the *LMO2* locus region for CBFB in murine thymocytes (A), RUNX3 in murine CD8+ SP T-cells (B) and RUNX1 in Jurkat cells (C). Elements previously described to be involved in the regulation of *LMO2* expression are indicated. Binding for CBF can be detected at element -25. A magnification of the region corresponding to element -25 is shown. Diagram of element -25 as previously. While binding of CBFB can be detected at both modules of element -25, RUNX1 and RUNX3 are preferentially bound to the 5‘ T-cell repressor module of element -25 (location of conserved RUNT motifs is shown in grey). D) T-cell repressor activity depends on a conserved RUNT motif. Conserved RUNT and LEF-1 motifs present in the T-cell repressor module of element -25 were mutated (5’ mRUNT -25 el. and mLEF1–25 el., respectively). Mutated constructs together with *LMO2* intermediate promoter element (mdp) were stably transfected in Molt4 T-cells and luciferase activity was measured. Mean and standard error of the means (SEM) for a representative experiment of at least two independent stable transfections (each one performed at least in triplicate) are shown. Values are expressed relative to empty vector, pGL2 basic. **P < 0.01; ***P < 0.001; ns, not significant.

All cell lines were stably transfected by electroporation as previously described [37]. G418 was added 24 hours post transfection and resistant cells were assayed 14 days later. Transfection experiments were performed at least in triplicate and at least on two different occasions. Results are shown as mean and standard error of the mean (SEM). Comparison among two groups was performed by t-test (Fig 1B and 1C). Comparison among more than two groups was performed by one-way analysis of variance followed by post-hoc analysis with the Bonferroni test for selected pairs of columns (Fig 1A) or Dunnett's test (Figs 2, 3D and 4D) to evaluate the significance of the differences between two groups. Statistical significance was assumed when P < 0.01.

### Display of ChIP-Seq traces

ChIP-Sequencing traces were retrieved from the publicly available compendium of Next Generation Sequencing experiments Codex (http://codex.stemcells.cam.ac.uk/) [38] and displayed in UCSC genome browser (http://genome.ucsc.edu/).

## Results

### Element -25 acts as a cell-type-specific enhancer

Element -25 consistently directs staining to foetal liver cells in transgenics and shows strong enrichment for active chromatin marks in the multipotential hematopoietic progenitors HPC7 and myeloid progenitors 416B [29]. Element -25 also displays strong enhancer activity in the myeloid progenitor cell line 416B in stable transfections [29]. To investigate its regulatory function in detail, element -25 in conjunction with each promoter element of *LMO2* (proximal, intermediate or distal) was inserted into the pGL2 basic luciferase vector and stably transfected into 416B cells. Of note, an extended version of the proximal promoter element (pPex) reported to provide stronger expression was used [29]. We found a consistent increase (over 10 fold) of luciferase activity of all 3 promoters in the presence of element -25 (Fig 1A), indicating that element -25 confers promoter-independent enhancer activity in 416B cells.

To investigate potential context-dependent activity of this enhancer, stable transfections were performed using the element -25 in collaboration with pPex (the most active promoter element, data not shown) in endothelial (MS1) and erythroleukaemia (F4N) cell lines, both of which express *Lmo2*. Element -25 did not exhibit any activity in either of these 2 cell lines (Fig 1B). These results suggest that element -25 acts as a cell-type-specific enhancer that controls the expression of *LMO2* in multipotent myeloid progenitors.

### Dual myeloid enhancer and T-cell repressor activity of element -25

Our data indicates that element -25 displays enhancer activity in specific cell types. Previous work from Hammond et al described a specific T-cell repressor region 2.5 kb upstream of the distal promoter element (dp) of *LMO2* [39]. Interestingly, this specific T-cell repressor region is contained within element -25. *LMO2* is not expressed in mature T cells hence we investigated the activity of element -25 in the T-ALL cell lines Jurkat and Molt4. Jurkat is a non-*LMO2*-expressing cell line while Molt4 expresses *LMO2* in the absence of a translocation involving *LMO2*. We found that a minimal version of the SV40 promoter directed low reporter expression in Jurkat cells and we decided to use the intermediate promoter element (mdp) in the cell line Molt4 since our group previously showed high activity of this element in this cell line [27]. We then performed stable transfections of the element -25 together with minimal SV40 or intermediate promoters in Jurkat and Molt4 T-cells, respectively. In line with the report from Hammond et al. [39], we found a consistent 80% repression inferred by element -25 in both cell lines (Fig 1C). These results indicate that element -25 shows a promoter-independent repressor activity in T-cells. Promoter-independent activity seems critical for genes such as *LMO2* where the gene locus contains three distinct promoter elements. Taken together, our results show that element -25 contains a cell-type-specific dual activity as both myeloid enhancer and T-cell repressor.

### Element -25 contains myeloid enhancer and T-cell repressor modules

To further understand the dual function of element -25, we produced three deletion constructs (Fig 2A) and analysed the effect of these deletions on the activity of element -25. To this end, we performed stable transfections of the different versions of element -25 together with the proximal or intermediate promoter elements in 416B and Molt4 cell lines, respectively (Fig 2B and 2C). When the central part of element -25 (445–1388 bp) was removed (del -25), we observed that both myeloid enhancer and T-cell repressor activities were completely abolished. To further dissect the element, a 5’ fragment (445–878 bp) was deleted (5’del -25); of note, this fragment included the T-cell repressor region previously described [39]. This deletion did not have any effect on the enhancer activity in 416B myeloid cells but completely abolished the repressor effect of this element in Molt4 T-cells. Finally, a fragment located in the 3’ region of element -25 (878–1388 bp) was deleted (3’del -25). In this case, deletion completely abolished enhancer activity in myeloid cells but did not have an effect on the repressor activity in T-cells. Taken together, our results show that element -25 comprises 2 juxtaposed independent modules that act as repressor and enhancer elements in a cell-type-specific manner.

### The 3’ myeloid enhancer activity of element -25 is dependent on GATA binding

To determine which transcription factors could be involved in the enhancer activity of element -25, comparative genomic analysis was performed. We produced a multi-specie sequence alignment for the 3’ region of element -25 (S1 Fig) and identified highly conserved transcription factor consensus binding sites in this region. Several potential transcription-factor binding sites that were highly conserved between species were identified using *TFBSsearch* [35] and *TESS* [36], comprising putative binding sites for Interferone Response Element 1 (IRF1) as well as E-twenty six (ETS), RUNT and GATA.

Our previous studies showed that ETS and GATA2 factors are bound to element -25 in 416B myeloid progenitors [29]; however, in order to gain insight into which binding motifs were actually occupied in this region, we interrogated a DNAseI genome-wide dataset performed in 416B cells [40] (Fig 3A). The most prominent DNaseI hypersensitive (DHS) region within the *Lmo2* locus in 416B cells can be detected in the genomic region corresponding to element -25, specifically in the region corresponding to the 3’ enhancer module of the element. A more detailed look at this module shows that there is a protected region between 2 highly hypersensitive sites that colocalises with the putative RUNT and GATA motifs. This pattern of DNaseI cleavage represents the classical pattern seen for regions that are strongly bound by transcription factors [41]. Investigation of recently published ChIP-Seq datasets furthermore supported GATA2 and RUNX1 binding to the myeloid enhancer moiety of this region in haematopoietic progenitors (HPC7) [42] and human CD34+ peripheral blood cells [43] (Fig 3B and 3C).

In order to test the importance of the RUNT and GATA motifs for the myeloid enhancer activity of element -25, we mutated the highly conserved RUNT (3’ RUNT) and GATA motifs present in the myeloid module of element -25 and then analysed the effect on luciferase activity in myeloid progenitors 416B. While the mutation of the RUNT motif did not reduce the activity of the promoter, mutation of the GATA motif drastically reduced the myeloid enhancer activity of element -25 (Fig 3D). Taken together, these results show that although the 3’ module of element -25 is bound by RUNX1 and GATA2 in human and mouse progenitors, the specific enhancer activity in myeloid progenitors only depends on the GATA motif.

### 5’ T-cell repressor activity depends on RUNT consensus binding site

In a similar way, we performed multi-specie alignments for the 5’ T-cell repressor module of element -25 (S1 Fig). The 5’ T-cell repressor shows less homology than the 3’ enhancer region revealing only highly conserved motifs for Lymphoid enhancer-binding factor 1 (LEF1) and RUNT proteins. During normal T-cell development, *LMO2* is expressed at the double-negative (DN) stage but is progressively down regulated during T-cell development. Although *LMO2* is expressed at fairly high levels in DN1 cells, its expression is severely reduced at DN2 stage and is barely detectable at the stages DN3 and DN4, double-positive (DP) and single-positive (SP) CD4+. *LMO2* cannot be detected in CD8+ SP cells [44].

RUNX1 and RUNX3 are important at different stages of T-cell differentiation. Both proteins can interact with the CBFb protein to constitute the so-called Core Binding Factor (CBF). We interrogated a previously published dataset for binding of CBFb in thymocytes [45]. Strong binding can be detected in the region corresponding to the repressor module for CBFb (Fig 4A). Of note, CBFb binding was also detected at the myeloid moiety of the enhancer, very likely due to the mixed population present in the sample. RUNX3 is known to act as a repressor of CD4 in CD8+ SP cells. We also processed and interrogated a dataset for RUNX3 in CD8+ SP cells [46] (Fig 4B) where strong binding for RUNX3 specifically at the 5’ repressor module can be detected. In a similar way, we interrogated a previously published dataset for RUNX1 in the Jurkat T-cell line [47], which does not express *LMO2* (Fig 4C). Like in CD8+SP cells, strong binding for RUNX1 can be detected at 5’ repressor module of the element -25. These results strongly suggest that the highly conserved RUNT motif found in the repressive module could be responsible for the repressor activity of this element.

We then decided to explore this hypothesis. We generated a version of element -25 where the RUNT motif located in the 5’ region was mutated and then we evaluated the effect in luciferase assays in Molt4 cells. We found complete abrogation of T-cell repressor activity by removing the RUNT binding motif at position 735bp (5’ RUNT), whereas activity remained unaffected after mutation of the LEF binding motif at position 549bp (Fig 4D). Our mutational analysis suggests therefore that the 3’ myeloid enhancer relies mainly on the GATA binding motif at position 1091bp whereas the 5’ T-cell repressor activity requires the RUNT binding motif at position 735bp (5’ RUNT).

## Discussion

We previously showed that the *LMO2* proximal promoter element drives *LMO2* expression in endothelial cells as well as in haematopoietic cells by transient transgenic mouse assays [28, 29]. However, cooperation with additional regulatory modules, such as element -25, was required for robust expression in haematopoietic cells [29]. In our previous study, we described that element -25 was important for *LMO2* expression in foetal liver cells, which comprises both erythroid and myeloid progenitors. In this study, we performed a detailed functional characterisation of element -25. We identified conserved transcription factor regions and provided functional demonstration of the effect of their deletion and mutation using stable transfection of haematopoietic cell lines. Moreover, we took advantage of previously published next generation sequence experiments and mined publicly available whole-genome transcription factor binding datasets to further support our results. Our current study reveals a dual cell-context dependent function of element -25. We identified 2 juxtaposed modules within element -25, one located in the 5’ region that acted as a repressor in T-cells and one located in the 3’ region that conferred enhancer activity in myeloid progenitor cells.

The spatial clustering of a myeloid enhancer and T-cell repressor may facilitate rapid “on-off switching” during the transition from a haematopoietic stem-progenitor to a T-cell lymphoid regulatory program. One potential mode of action would be that the close proximity of the 3’ myeloid enhancer and 5’ T-cell repressor module may accelerate the deployment of enzymes associated with the epigenetic machinery to the adjacent region of opposite functionality. It has been shown before that chromatin states can spread from an initial nucleation event (reviewed in [48, 49]). It will be intriguing therefore to investigate whether similar clustering may constitute a general characteristic of critical regulatory elements of developmental genes required for a transition from stem/progenitor cell to a more differentiated program.

The activity of the 3’ myeloid enhancer is strongly dependent on a highly conserved GATA site that is bound by GATA2 in mouse and human haematopoietic progenitor cells. Surprisingly, even though there is a highly conserved RUNT motif in this part of element -25, the myeloid enhancer activity was not only independent on this motif but the activity increased following the mutation of this motif. However, strong protection within the DHS could be detected in the region corresponding to the RUNT motif in 416B myeloid progenitors which indicates that this site is occupied in these cells and RUNX1 is also strongly bound to this region in progenitors (Fig 3). These results suggest that RUNX1 may exist in different complexes in myeloid progenitor cells that can have either activating or repressing enhancer activity. Direct interaction between RUNX1 and GATA2 and recruitment of RUNX1 in the absence of RUNT motif by GATA2 has been previously reported [42]. It is then reasonable to assume that RUNX1 recruitment to the myeloid enhancer module of element -25 is still important, although this may be mediated by GATA2 when the RUNX site is mutated. RUNX1-activating complexes would then contain GATA2 and mainly depend on GATA2 binding. A RUNX1-fusion transcription factor complex has been recently described in the Kasumi-1 myeloid cells [50, 51]. In these cells, the RUNX1-fusion complex mainly acts as a repressor [51] and lacks GATA2 [50]. In Kasumi-1 cells, coexistence of RUNX1 active and repressive complexes ensures fine regulation of the expression of multiple genes [51]. Interestingly, the strongest binding event in the *LMO2* locus for AML1-ETO containing-transcription factor complex (of which LMO2 is a key component) in Kasumi-1 cells (that express high levels of *LMO2*) is located at the 5’ enhancer region of element -25 [51]. Similarly to our results, treatment of Kasumi-1 cells with shRNA against the fusion protein resulted in a slight upregulation of the expression of *LMO2* [50].

The 5’ T-cell repressor module of element -25 that we describe overlaps with a previously described repressor element in *LMO2* [39]. In contrast to our results, the previous study reported that the RUNT motif is not important for the repressor activity. These discrepancies may be due to at least two reasons. Firstly, we used Molt4 cells as a model in contrast to the previous study which used Jurkat cells. The use of Molt4 cells is supported by our previous observation that Molt4 cells express *LMO2* at higher levels than Jurkat cells [27] in the absence of any known translocation involving the LMO2 locus. Secondly, the manner in which luciferase assays were performed differed in both studies. In our study we performed stable transfections in contrast to that of Hammond et al. which used transient transfections. Only the use of stable transfection assays allows a comprehensive evaluation of any transcriptional effects associated with integration into chromatin, which is known to be pivotal for multiple aspects of transcriptional repressor activity.

### Critical role of RUNX factors in down-regulation of *LMO2* during T-cell development

*LMO2* down-regulation is known to be critically required for terminal differentiation of T-cells. In murine models, enforced expression of *Lmo2* in thymocytes blocks differentiation at the double negative stage and, eventually progression to a T-cell lymphoproliferative disease takes place [9–13]. On the other hand, up-regulation of *Runx1* is essential at late double negative stages and, in *Runx1* deficient mice, development of T-cell lymphoma has been reported [52]. The role of RUNX proteins in T-cell development has been extensively studied, especially in the context of the CD4/CD8 specification process. Thus, it is known that a 434 bp repressor located in the CD4 locus is responsible for the appropriate spatial and temporal expression of CD4 during T-cell development. This repressor is bound by RUNX1 at early stages of development (DN) and by RUNX3 in CD8+ SP cells and requirement for these factors in CD4 silencing was confirmed using transgenic mice deficient in *Runx1* and *Runx3* at the appropriate stages [53, 54]. We show in our study that CBF binds to the 5’ T-cell repressor region in thymocytes and CD8+ SP cells, similarly to the CD4 repressor. Our results therefore support a regulatory model, in which the up-regulation of RUNX proteins during T cell differentiation is required for the down-regulation of *LMO2* mediated by CBF binding to the 5’ T-cell repressor module of element -25. According to this model, translocation or functional abrogation of the 5’ repressor within element -25 would lead to failure of *LMO2* down-regulation in a lymphoid cell context and to ectopic *LMO2* expression. Our results provide a molecular link between RUNX proteins and *LMO2* and underline the importance of RUNX transcription factors not only in the specification of haematopoietic stem cells but also during maturation of T-cells [55].

### Segregation of the RUNX-dependent 5’ T-cell repressor module as a main mechanism for ectopic *LMO2* expression in TCR-LMO2 rearranged T-ALL

The mechanisms deregulating *LMO2* expression are numerous and more varied than initially assumed. Nearly 50% of human T-ALLs that lack chromosomal lesions involving LMO genes present overexpression of *LMO2* or its family member *LMO1* [56]. Recently our group reported activation of a novel intermediate promoter element at –11.8 kb upstream of the proximal promoter in a number of cytogenetically normal T-ALL patients as a possible mechanism for ectopic *LMO2* expression [27]. In the case of chromosomal abnormalities that involve *LMO2*, including translocations and deletions, the presence of activating elements in the vicinity of *LMO2* as a result of the abnormality was believed to be the cause. However, it has been suggested that removal of the T-cell repressor region was the truly activating mechanism [25, 26, 39]. Most of the studied *LMO2* break-point regions in rearranged T-ALL patients clustered in the region between the T-cell specific repressor and the proximal promoter element (~1.7 kb) and break-points contained within this region were very strongly associated with high *LMO2* expression levels [25, 57–62]. The factors that govern the occurrence of recurrent translocations in this area are still unknown. Two potent cryptic recombination signal sequences that resemble TCR/Ig recombination signal sequence have been identified in this region. Interestingly, one is located within the 3’ enhancer here described and a second one is located 370 bp downstream of element -25 [25]. The existence of an enhancer element located immediately downstream of the repressor that is active at the early stages of T-cell development could potentially provide chromatin accessibility for RAG mistargeting which could explain the recurrent oncogenic translocations into this region [25]. It may be the dual nature of the -25 region therefore, containing both activating and repressing activities, that makes it a particularly vulnerable target for oncogenic translocations that cause ectopic expression of *LMO2*.

## Supporting Information

S1 Fig Multi-sequence alignment of element -25. Homologous genomic sequences of the element -25 were downloaded from *Ensembl* for human, mouse, cow, dog and cat, aligned using *multi-Lagan* and displayed using *Genedoc*. Regions corresponding to the 5’ T-cell repressor and 3’ myeloid enhancer are indicated. Highly conserved sequences are depicted in black and candidate transcription factor binding sites are indicated. Arrowheads indicate the previously described T-cell repressor-region [39].
(PDF)

S1 Table Primers used for amplification of human LMO2 regulatory elements. (DOCX)

S2 Table Primers for site-directed mutagenesis of LMO2 element -25. (DOCX)
