# Supplementary material for: The LMO2 -25 Region Harbours GATA2-Dependent Myeloid Enhancer and RUNX-Dependent T-Lymphoid Repressor Activity
Source: PLoS One. 2015 Jul 10;10(7):e0131577. doi: 10.1371/journal.pone.0131577 (PMC4498896; doi:10.1371/journal.pone.0131577)
Supplement: S1 Fig — Homologous genomic sequences of the element -25 were downloaded from Ensembl for human, mouse, cow, dog and cat, aligned using multi-Lagan and displayed using Genedoc. Regions corresponding to the 5’ T-cell repressor and 3’ myeloid enhancer are indicated. Highly conserved sequences are depicted in black and candidate transcription factor binding sites are indicated. Arrowheads indicate the previously described T-cell repressor-region [39]. (PDF) [file pone.0131577.s001.pdf]

```
human : GGCCTAAAACCTTCTAGAGGGTCTCACTCTAAAGATAACCC-AAAGGTCTCACAGCTTCCTGGTTGC :  66
mouse : AGCCTGGAA-CTTCTCCAGGATCTTATTCTAAAGACACCCCTCAGGACCTTGCTGCTTTCTGGCTAC :  66
cow   : AGCCTAAAACTTTCTGCAGGGTGTTACTCTAAAGATAACCCAAAAGGTCTCATAGCTTCCTGGTTGT :  67
dog   : GGCCTAAAACCTTCTGCAGGGTGTCACTCTAAAGATAACCCAGAAGGTCTCCTAACTTCCTGGTTGT :  67
cat   : AGCCTAAAACCTTCTGCAGGGTATCACTCTAAAGATAACCCA-AAGGTCTCATAGCTTCCTGGTTGT :  66

human : AAAAACGACA---ACAGGAGCAGGAATTGAGACCTAAAGATCATTTGGGTAGAAATGAGGAAAGAAA : 130
mouse : TCAGA---TGTGTACAGTAGCAGGACTTGAGACCTGAAGATCAGTGGGGTAGAAATGAGGAAGGAAA : 130
cow   : TAAAACAACAACTACAGTAGCAGGAATTGAGACCTAAAGATCAGCAGAGTAGAAATGAGGAAGGAAA : 134
dog   : TAAAACAGCAACTACAGTAGCAGGAATTGAGACCTACAGATCAGTGGCGTAGAAATGAGGAAGGAAA : 134
cat   : GAAAACAGCAACTCCAGTAGCAGGAATTGAGACCTACAGATCGGTGGGGTAGAAATGAGGAAGGAAA : 133

human : TGACATGTTAAATGATGATTTT-TCCCTAAC-TTGCCTTGTTCTTTCTAGATGTGGCTCAAGAACTT : 195
mouse : TGACATGTTCCATGACTAGTG--TCCCCAGCTTTGTCTTGCTCTTTCTAGATACAGCGC-AGACCTC : 194
cow   : TGGCATGTTGAATGACTATTTT-TCCCCAAC-TGGCCCTATTCTTTCTAGATATAGTCCCAGAACTT : 199
dog   : TGGCATGTTAAATGACTCTTTCCTCCCCAGC-TTGCCCTGTTCTTTCTAGATATAGCCC-AGAACTT : 199
cat   : TGGCATGTTAAATGACTATTTCCTCCCCAGC-TTGCCCTGTTCTTTCTAGATATAGCCC-AGAACTT : 198

human : GCAGAGTTATGGTGGGATAATTCGACTGATCAAATGCTGAAAGT---TTTCCTATCGACTTCAAGGC : 259
mouse : ACATGGCTATAGTGTACTTGTCTGGC----CAAATGTGGAAAGGTTTCTTTCTATCTGTTTCAAGGC : 257
cow   : GCATAGTTACAGTGGGAGAATTTGATCGATCAAAGGCTGAAAGT---TTT-CTGCCAAGGTTCAGGC : 262
dog   : GAATAGTTACAGTGGGATAATTTGAT----CAAATGCTGGAAG----TTTTCTATCAATTTCAAGGC : 258
cat   : GCATAGCTACAGTGG--------GAT----CAAATGCTGAATG----TTTTCTATCAATTTCAAGGC : 249

human : TGTTCAAATCTCTATGTACTTTGAAGTCTGAGGCACAAGATAATTTCCCAGAGATTCATGTTTCTAT : 326
mouse : TGCTTAAATCT-TATACGTTT-------TGAGATGCAATAGGATTCCCTGGAAATTCATATGTCTCT : 316
cow   : TGTTCAAAACTTGAAGTACTTCAAAGTCTGGGGTGTAGTGGAATTCCTAAGAAATTTATGCCTTTAT : 329
dog   : CGTTCAAATCTCTATGCACTTTGAAGTCTGGGGTGCAGTGGAATTCCTGGGACATTTGTGCTTCTGT : 325
cat   : TGTTCAACTCTCTATGTACTTTGAAGTGT-GGGTGCGGTAGAATTCCTGGGACATCTATGCTTCTAA : 315

human : CGATGCTAGGCTGGGCTGACACAGATAACCCCTCAAGAGGCCTTTC-CGAACTGCTGTATAAACTGT : 392
mouse : CTAAATTGGGCTGGTTTGGCATTGATCTCTCTTC-----------CCAAAGACATTGT--------- : 363
cow   : CTCTGTTAGGCTGGTTTGACACAAATACCCTCTTAGGAGGCCCTTCGCAAGCCACCATGTAAATTCT : 396
dog   : CCCTGCTAGGCTGATTGGACACGGATACCCCCTCGGGAGGCCTTTCCCCAGCCCCTGTGTGAACTGC : 392
cat   : CTCTGCTGGGCTGATTGGACACAGATTCCCCCTCAGGAGGCCTTTCCCAAGCCACTGTGCAAACTGT : 382

human : CCTGGGTCCAGCAATAAAGCAACTGTGTCATTATAGTTCTCTGCCCTTCAGAAGATCTGATCTTAAT : 459
mouse : --AGGGCCCAGCCATAAAGAAACTATGTTCTTAAGGCCCTCTGCCCTTGAGGGCAGAT--------- : 419
cow   : CCTAGGCCCA-CAACGAAG----TGTGTTCTCGTAGCCTTCTGCCCTCC-GAGGATCTCATTTTAAT : 457
dog   : C-CTGGGCCAGCGGTAAAGCCTGCGTGCCGTTACAGCCCTCTGCCCTTCCAAAAATCCTAGT----- : 453
cat   : C-CTTGGCCAGCAGTGAAGCCAGTGTACCATTACCCTTCTCTGATCCTCAGAAGATATTAAC---TG : 445

human : ACCCTTCTCTTCTTTTTGTCC--CTCTGATTTCATCAAATTTGTTGCCATTCT-------------- : 510
mouse : --------CTGATCTTAATGTACTCCCAACTCCATCTGATCTGTTGTAACTAT-------------- : 464
cow   : TCC---TGCCTCTTTTTGTCCTTTTCTGGTTTCATTAAATTCGTTGTCACTTTGGCTCTTTTCTTTT : 521
dog   : GCCCTCCGCTTCTTATTGTCCATTTCTGGTTTCATCAAATTTGCTGCCGCTTTGC------------ : 508
cat   : TACCTTCACTACTTATTGTCCC-------TTTCATCAAA-TAGCTGCCACTTGG------------- : 491

human : ------------------------------------------------------------------- :   -
mouse : ------------------------------------------------------------------- :   -
cow   : TTTGTTGTTTTCATTTTTATTGGCATATGATTGCTTTCGAAGTTGTGTTAGTTTCTACTGTACAGCA : 588
dog   : ------------------------------------------------------------------- :   -
cat   : ------------------------------------------------------------------- :   -
```

5’ T-cell repressor module within element -25

```
human : -------------------------------------GGGTGTTTCTCCTTCTCTCCCCGACTTTTT :  540
mouse : ---------------------------------------TTCTCTCCCCAATTCT---------TTC :  483
cow   : AAGTCAGCTATATATATACATATATCCCCTCTTTTTTGGATCTTTCTCCCTCTCTCTTCCCATTTTT :  655
dog   : ---------------------------------------TTCTTTCTCAGTCTCTCCTCACATTTTT :  536
cat   : ---------------------------------------TTCTTTCTCCCTCTCTCACCGCATCTTT :  519
```

LEF

```
human : CTACTTCAGATCAAAGAACTTCCCCTTTTTCCCACTTTTGGATTTGCGCCCTGGAGGACAACGCAAG :  607
mouse : CTACTTCAAATCAAAGAACT------CATCCTTATGACTGGATTCACCACCTTGAGGGCAATGCAAG :  544
cow   : CTATTTCAGTTCAAAGGAGCTCTCCATTTCCTCACTCCTGAAATTACTCCCTGGAAGGCAAATCCAG :  722
dog   : CTACTTTAGATCAAAAGACTTCCCCATTTCCCCACTTCTGGATTTGCTCCCTGGAGGGCAAAGCGAA :  603
cat   : ATACTTCAGATCAAATGTTTTGCCCACTTCCCCGCTTTCGGATATACTCCATGAAGGGCAAAGCAAG :  586
```

```
human : GTTAACTTCCCTCCCACCTCCTCTTGTAGGCACACAGGGTGCCCCTTAGCC---------------- :  658
mouse : GTCCGGCTTGTTCCTGCCTCCCTGTCCAGGCA-TCAGGGCTCCTAAC-------------------- :  590
cow   : ------TGATTTCCCATCTCCTTTTCCAGGCACACAGGGTGCTCCTTAGCTG--------------- :  768
dog   : CCT------CTTTTTCCAGGCACACAGGGTGG-AGGGAG-----------ATGCGGCGGGGGCGGGG :  652
cat   : GCTAAG-TCCTTCCTCCGTCCTTTCCGTGTCA-ATTGGGCTCTCCTTAGCGTTGGGCAGGGGTGGGG :  651
```

```
human : ---AGCGAAATCTCCGC-----------GCCCGAGATCCTCTCACAATTTCAGGCT-GGTGAATTGT :  710
mouse : -TTTACAAAATCTGCACC----------CTCAGAGCTACCCACAGAGCTGCAGGCTCAAAGCGGTAA :  646
cow   : ---AATAAGATCCCCACCCCAGCCCCCACCCCGAGAG-CTCTGGCAATTTCAGGC--------TTGT :  823
dog   : AGTGGGG-TATCTCCA-C----------CCACTGTGGGCCCTCACGGCTTCAGGCTC-CCGAGCTGG :  706
cat   : TGGGGGGAGATCTCCACC----------CCGCTGTGAGCTCTCGAGATTTCAGGCAC-CTGAGCTGG :  707
```

5’ RUNT

```
human : AGTAGGAGACATGGTTTAGTTTGATGTGGTTAAGTCAGTCATCATGGCATCTTCGTACCCTCAGCCC :  777
mouse : CAAAGAGAAAGGGGGTTGGTTTGATGTGGT-TAGCCTGCCACCCCTGCCTTTTCATGGTCCTGGCTC :  712
cow   : AGCAAGAGTAAAGCTTTAGTTTGATGTGGTTAAACCAGTCATCACAGTGTCTTCAGACTCTCAGCCC :  890
dog   : GGCAGCAGAAAAGGTCTAGTTCGATGTGGTCCCATCTGCCATCACTGCGTCCTGGTCCTCTCTGTCC :  773
cat   : GGCAGGAGAAAAGGTCTAGTTTGATGTGGTCCCGTCAGCCATCACTGTGTCCTCATACTGTCTGCCC :  774
```

```
human : CAAACCGCTCTCCTACTCATCACCCTCACTTT-CATTTCTTTGGAT------------TATCTTTCA :  831
mouse : TAAACCACTCTCCTGCTCACCCCTCAGCCTGCACTGGGCTCTGGGGATGAATGCATGATAGACTTCA :  779
cow   : CAAACCACTCTGGGGTACATCACCCTCACTTTCCATTTCTCTGGAT------------TATCTTTCA :  945
dog   : CCAGCCCCTCTCCTGTGCACCCCCCTCATATTCCACGGCTCTGG----------ATTATAGCTTTCC :  830
cat   : CAAACCTCTCTCCTAC-CGCCACCGTCACCTTCCAGTTCTCTGG----------GTTAGAGCTTTCC :  830
```

```
human : TCGGCTTACCACCAGCCTTCACAGCCTCACCCAAGGCTGAAGC-AGGGCTAGCAGGGTGGTGCGT-- :  895
mouse : CAGCCTCACCA----------------------GAGACTGAAGCAGGACCCACGGGTGGGCAGGGGC :  825
cow   : TCTGTTCACCACTAACCCTCACAGCCTCAACCAAGGCTGAAGCGGGGCCAGCAGGGTGGTACGC-- : 1010
dog   : TCTGTTCACCAC--------------TCCT-------CAAAGCAGGGGCCAGCAGG----------- :  865
cat   : TCTGTTTACCAC--------------TCCTGGGAGGCTAAAGCAGGGGCCAGAGGGGAGATGCA--- :  880
```

3’ myeloid enhancer module within element -25

```
human : ---TTCCTTCCCTGGTTGAGCTGGTGACTGCTGTGGGT-AAGCAGGTCCAATGTGAACTCAATTTTA :  958
mouse : ACTATCATTTTTTCCCTGAATTCTTGGGTGCTTAGGCC-GGCTCAGCCTCGG-------------GA :  878
cow   : ---TTCCTTTCTTGGTTGAAATACTAACTGCTGTGGCTAAAGCCATTCCCTTTCGGACTCA---TTA : 1071
dog   : ----------------TGAACTGCTGACTGCTGTGGCT-AAGCCATCCCAGGTCCTGCTCAT---GA :  912
cat   : --TTTCCTTTCCTGGCTAAACTACTGACTGCTGTGGCT-AAGCCATTCCTGGTCCTGCACAT---TA :  941
```

```
                         IRF1                                      ETS
human : CATT------GAGAAAGAGAAAGCAAGAGTGAGT--GGGCTGCTAAGACTGTTGCTTCATGGTTTCC : 1017
mouse : TTGGGACCAAGAGAAAGAGAAAGTGAGAGCAAGC-AGTGGAGCAAAGGCTGTTGTTACACAGTTTCC :  944
cow   : CAGGGAACAAGAGAAAGAGAAAGCAAGAGTAAGC----GCTGCAAAGACTGCTGTTACATGGTTTCC : 1134
dog   : CAGGGAACAAGAGAAAGAGAAAGCAAGAGGGAAT-GGGGCTGCAAAGACTGTTGTTGCATGGTTTCC :  978
cat   : CAGGGAACAAGAGAAAGAGAAAGCAAGAGTGAGCGGGGGCTCCAAAGACTGTTGTTGCATGGTTTCC : 1008
```

```
                                     ETS                        3’ RUNT
human : CCTAAGCCCCAGAAATAGAAAGGCAGACTTCCGACTGGCAGAGCCCGTATGCTAACCACCAGACCAC : 1084
mouse : CTTCAGCCCCAGAAATAGAAAGCCAGACTTCCGACTTGCACAGCCCCTGTGCAAACCACTAGACCAC : 1011
cow   : CTTAAGCCCCAGAAATAGAAAGCCAGACTTCCGACTTGCAGAGCCTCTGTGCGAACCACCAGACCAC : 1201
dog   : CTTAAGCCCCAGAAATAGAAAGCCAGACTTCCGACTTGCAGAGCCGCCACGCAAACCACCAGACCAC : 1045
cat   : CTTAAGCCCCAGAAATAGAAAGCCAGACTTCCGACATGCAGGGCCGCTGTGCAAACCACCAGACCAC : 1075
```

```
                 GATA
human : ACTGCCAGATAAGAAAAATGTTCGATTGTGTCATACTTCCCTCATTGGCTCCAGTGTCTGAAAATGG : 1151
mouse : ACTGCCAGATAAGAAAAATGTTAGATTGTGTCATACTTCCTTCATGGGCTGCAGCTTCTGCAAATGC : 1078
cow   : ACTGCCAGATAAGAAAAATGTTCGATTGTGTCATATTTCCTTCATCAGCGCTAGTTTCTGAAAATGG : 1268
dog   : ACTGCCAGATAAGAAAAATGTTAGATTGTGTCATACCTCC------AGCCCCAGTTTCTGAAAATGG : 1106
cat   : ACTGCCAGATAAGAAAAATGTTAGATTGTGTCATACTTCC------AGCTCCAGTTTCTGAAAATGG : 1136
```

```
human : CCCTGAATTTCAGAGAGTCTTACCATGACCCAGGGATTTAGAGAGGATCGAAGAGGGA--------G : 1210
mouse : CCCTGTGTGTCAGAGAGTCTAAGGAA-----TGAGCATCTAAGGGGCTAGATGGAGAA---------A : 1132
cow   : CTCTGAGTTTCAGAGAGCCCGGCCATGATCCTAAGATTAAGAGAAGATGAAAGAGGAA---------A : 1327
dog   : CCCTGAATGTCCAGAGCTCCGCCATGACCCTAAGATTCAGAGAAGGCGGAGGAAGGT---------G : 1165
cat   : CCCTGAATGTCAGAGAGCTTCACCACGATTCTAAAA-TCAGAGAAGATGGAAGAGGGTGGGGAGTGG : 1202
```

```
human : GACAAAGGAATCAGGCACCTTCTCTCTCTCTTTTCTGGGG--TAAATTTATTTTCAACCACAAAAGT : 1275
mouse : GCCAACAGAGACTGT--------TGTCTCTCCTTTCTGGTT-ATAGGTTCATGTGTAACCACTAAAGG : 1191
cow   : GAAGAAGGGATCAGA--------TCTCTTTCTTTTCAGGTA--TAAGTTTATTTTCAACCACATAAGT : 1385
dog   : GACAAAGGGATCAGG--------CCTCTCTCTATTCTGGTATCTGAATTTATTTTCAATCACAGAAAT : 1225
cat   : GGCCAAGGGATCAGG--------CACCTCTCTATCCTGGTA-------TTAATGTCAGCCACAAAAAA : 1255
```

```
human : AAAGGAAAGAAAAAGTCACAACAGATGAGTT------------------------------------- : 1306
mouse : AAAGGGGAG-----CTCACAAAAGCATG---------------------------------------- : 1214
cow   : AAAGGGAAGGGAAGATTATGAGAGATTAGAC------------------------------------- : 1416
dog   : AAAGCAAAGCAAACATCATGAAGTGTTGGT-------------------------------------- : 1255
cat   : CAAAGAAAA-AAATATTATGAAAGATTAGTCATGAGCTTTCTTTTTTATATAAAATTGCTAATGTT : 1321
```

```
human : ------------------------------------------------------------------- :    -
mouse : ------------------------------------------------------------------- :    -
cow   : ------------------------------------------------------------------- :    -
dog   : ------------------------------------------------------------------- :    -
cat   : TGTATTCATTTTTGAGAGATAGAGACAGGATGTGAGTGGGGGAGGGGCAGTGAGAGAGGGAGACACA : 1388

human : ------------------------------------------------------------------- :    -
mouse : ------------------------------------------------------------------- :    -
cow   : ------------------------------------------------------------------- :    -
dog   : ------------------------------------------------------------------- :    -
cat   : GAATCTGAAGCAGGCTCCAGGCTCTGAGCTGTCAGCACAGAGCCCTACGTGGGGCCCGAACCCATGA : 1455

human : ---------------------------------------------------------------ATGA : 1310
mouse : --------------------------------------------------------------AGCGA : 1219
cow   : ---------------------------------------------------------------GTGA : 1420
dog   : --------------------------------------------------------------CGTGA : 1260
cat   : ACCGTGAGATCACGACCTGAACGGAAGTCAGCCGCTTAACTGACTGAGCCACCCAGGCACCCCATGA : 1522

human : GCTAACTTTCTGATTTGCACATGTAA----------GAGAAAGAAATGAACTGGATATCATTAGACA : 1367
mouse : GCTAACTTAATTATTTGCATGTATGA----------GAGAGGAAAACTAATT--------------- : 1261
cow   : GCTAACTTT---ATTTGCAAAAGAAATTTTTTATTTGCAAGAGAAATGAATTGGACATCAACAGGCA : 1484
dog   : GCTAACTTTCTGAATTGCACGTACA------------AGGAAGGAATGAGCTGGATGGCAACACGTA : 1315
cat   : GCTAACTTTCTTAATCGCATGTACAA----------GAGGAGGAAATGAACTGGATATGAACAAGCA : 1579

human : TTGAGAAAGGGAGTCT-GCAAGCT---AGCAATGTATCTTCGGCCTTTTTGATTTACACGTGTTTAT : 1430
mouse : ---------GGAGTCT-GCTAGCTA--GCTCCTGTGCCCTTGACATTTTCAGTCCCCGTATACTTAT : 1316
cow   : TTGAGAAAGGGAGCCT-GCCAGTTTAAAAAAATGTGTCCTTGGTCTTTTCAATTCCCCTGTGTTTAT : 1550
dog   : TTGATGAAGGGAATCC-ACTAGCTT--AAAAACATCTCCTTGGCCTTTTCAGTTCGTGCGCGCTCAT : 1379
cat   : TTGAGAGTGGGAATCCAACTAA-----AAAAATGTCTCCTTGGCCTTTTCAATTTGTGCGTGTTCAT : 1641

human : TTA-TGAGTACCTACTGTGTGCCAGGCATGA--TGCTTGGTGCTGCCAATTCAAAGAGAAATAAGAC : 1494
mouse : TTATCAAGTACCAACTGGGTACCAGCTGCC---ACATTAGTACTGCAGATTTTTGGTG-CAGAACA- : 1378
cow   : TTATTGGGTGCCTGCTCTATGCCAAGGGCTG--TGCTTATTGCTGCAAATCCAAAGAG-GATGAGGC : 1614
dog   : GCATTGAGTGCCAACTGTATGCCAAGCACAGTTTGCTTGGCGCTACGGATTCGAAGAG-GATGGGAC : 1445
cat   : TTATTGAGTACCGACTGTATGCCAACCACG---TGCTTGGTGCTGTGGATTTGAAGAG-GACCATAC : 1704

human : ACCTCCTTGCAAAGTGAGAAGGCAGAACTAGATTCAGATTCACCTAGATTTCAACTCTTGTGCTCAG : 1561
mouse : ----------------GGGAGGCTGGCCCAAAGTCAGAATCACCTAGATTCCACCTA-TGGGCTCAG : 1428
cow   : ATCTGTTCACAGAACAAGGAGGCTGGACTAGATTCAGATTCACCTAGATTCCAACTCATGTGCTCAG : 1681
dog   : ATCATCGC--------AGGAGCCTGGACTATATTCAGATTCACCCCGATTCCAACTCTTGTGCTCAG : 1504
cat   : ATTGCAGA--------CTGAGGCCGGACTATATTCAGATTCACCCAGATTCCAACTTTTGTGCTCAG : 1763

human :   CACCACC CCACCATGGAAA AATTTTGCTT A TCT---CCCGGCGCAGCCGCGGCAGGCGTGT : 1625
mouse : --TACCACTGC-ACCATGGAAATAATTTCCCTTCATTCTTTCCCCAGAGCAGCCTCAGCAGGCTCCC : 1492
cow   : --CATCACCTCCACCACCAAAATAATTTTACTCCACTCT---CCCAGAGCAGACTTGGTGGGCACCT : 1743
dog   : --CATTACCTCCACCGTGGAAATAATTTTGCTTCGTTGT---CCCAGAGCAGCCCCGGCGGGCGCCT : 1566
cat   : --CACTACCTCGGCCATGGAAATCATTTTGCCTCGTTCT---CCCAGAGCAGTCTCCGCGGGCGCCT : 1825

human : GTGTTGTCAGACACTGCAGGAAGAGTGATGGCCTGGGTGTTGACAGGCTCCTAATTGGAAGGTAGGA : 1692
mouse : GTGTTGTCAGCCACTATAGGAAGAGTGATGGTCTGGGTGTTGACAGGCTCCTAATTGGAAGGTAGGA : 1559
cow   : GTGCTGTCAGCTGCTGCAGGACGAGTGATGGCCCAGTTACTGACAAGCTCCTAATTGGATGGTAGGA : 1810
dog   : GTGTTGTCAGCCGCTGCAGGAGGAGTGATGGCCAAGGTGTTGACAAGCTCCTAATTGGAAGGTACGA : 1633
cat   : GTGTTGTCAGCCGCTGTAGGAAGAGTGATGGCCGGGGTGTTGACAAGCTCCTAATTGGAAGGTAGGA : 1892
```

```
human : GGTGCCACCAGAGATCAGCTATAAAAGA-TATCTTTGGACTGGGGTGGGCTGCTAATGCTTGGAAGA : 1758
mouse : GGTGCCGCCAGAGATCAGCTATAAAAGT-TATCTTTGGATGGGGGTAGGCTGCTGATGCTCAGAAGA : 1625
cow   : GGTACC--------ATCAGCTATAAAAGGTTATCTTTGTACTAGGCTGGGCTGCTTATGCTCAGAAGA : 1870
dog   : GGTGCCGCCAGAGATCAGCTATAAAAGG-TATCTTTGGGCCGGGGTGGGCTGCTAATGCT--GCAAG : 1697
cat   : GGTGCCGCCACAGATCAGCTGTAAAAGA-TATCTTTGGGCCAGGGTGGGCTGCTAATGCT--GGAAG : 1956

human : GACTTGT-GGGTGAGGCCCGGTGAGCTGTCACCTCGGAATTGGG : 1801
mouse : GAATTAG-GGGTGAGGTGGGGTGAGCTGTCAGCCTGGGACAGGG : 1668
cow   : GACTTGGAGGGTGAGGCCTGGTGAGCTGTCAACCTGGGGTAGGG : 1914
dog   : AGACTCG-GGGTGAGGCCTGGTGAGCTGTCACCCTGGGATGGGG : 1740
cat   : AGACTCG-GGGTGAGGCCTGGTGAGCTGTCACCCTGGGAGGGGG : 1999
```
