# Supplementary material for: The LMO2 -25 Region Harbours GATA2-Dependent Myeloid Enhancer and RUNX-Dependent T-Lymphoid Repressor Activity
Source: PLoS One. 2015 Jul 10;10(7):e0131577. doi: 10.1371/journal.pone.0131577 (PMC4498896; doi:10.1371/journal.pone.0131577)
Supplement: S1 Table — (DOCX) [file pone.0131577.s002.docx]

**Table S1. Primers used for amplification of human *LMO2* regulatory elements**

| **element** | **forward primer** | **reverse primer** |
| --- | --- | --- |
| -25 | GGCCTAAAACCTTCTAGAGGG | GCCCAATTCCGAGGTGACAG |
| dP | GGAGAAGTAAATACAGGCTG | GAGTGGTCTCCCTTTGTGG |
| mdp | CCACCTGATACACCAGTCC | CCACTAGCTACTGCAAGTTC |
| pPex | GCAGTTTAACCGCAGGAGTCCG | TGGGGAGGGAGGCGG |
