# Supplementary material for: The LMO2 -25 Region Harbours GATA2-Dependent Myeloid Enhancer and RUNX-Dependent T-Lymphoid Repressor Activity
Source: PLoS One. 2015 Jul 10;10(7):e0131577. doi: 10.1371/journal.pone.0131577 (PMC4498896; doi:10.1371/journal.pone.0131577)
Supplement: S2 Table — (DOCX) [file pone.0131577.s003.docx]

**Table S2. Primers for site-directed mutagenesis of *LMO2* element -25**

| **binding site** | **sequence** |
| --- | --- |
| **5’ region** |  |
| **a) LEF site (pos 549)** |  |
| Wild type | CCGACTTTTTCTACTTCAG***ATCAAAG***AACTTCCCCTTTTTCCCACTTT |
| sense primer (*NdeI*) | CCGACTTTTTCTACTTCAG***ACATATG***AACTTCCCCTTTTTCCCACTTT |
| antisense primer | AAAGTGGGAAAAAGGGGAAGTT***CATATGT***CTGAAGTAGAAAAAGTCGG |
| **b) 5’ RUNT (pos 735)** |  |
| Wild type | GACATGGTTTAGTTTGA***TGTGGT***TAAGTCAGTCATCATGGCATC |
| sense primer (*PsiI*) | GACATGGTTTAGTTTGA***TGTTTA***TAAGTCAGTCATCATGGCATC |
| antisense primer | GATGCCATGATGACTGACTTA***TAAACA***TCAAACTAAACCATGTC |
|  |  |
| **3’ region** |  |
| **a) 3’ RUNT (pos 1080)** |  |
| Wild type | CCGTATGCTAACCACCAG***ACCACA***CTGCCAGATAAGAAAAATG |
| sense primer (*PvuII*) | CCGTATGCTAACCACCAG***CTGACA***CTGCCAGATAAGAAAAATG |
| antisense primer | CATTTTTCTTATCTGGCAG***TGTCAG***CTGGTGGTTAGCATACGG |
| **b) GATA (pos 1091)** |  |
| Wild type | CCAGACCACACTGCCA***GATA***AGAAAAATGTTCGATTGTGTC |
| sense primer (*XbaI*) | CCAGACCACACTGCCA***GTCT***AGAAAAATGTTCGATTGTGTC |
| antisense primer | GACACAATCGAACATTTTTCT***AGAC***TGGCAGTGTGGTCTGG |
